# A simple quality assurance test tool for the visual verification of light and radiation field congruent using electronic portal images device and computed radiography

**DOI:** 10.1186/1748-717X-7-49

**Published:** 2012-03-27

**Authors:** Christopher F Njeh, Blas Caroprese, Pushkar Desai

**Affiliations:** 1 Texas Oncology Tyler, Radiation Oncology Department, 910 East Houston Street, Tyler 75703, TX, USA; 2 East Texas Medical Center, Radiation Oncology Department, ETMC_Cancer Institute, 721 Clinic Drive, Tyler 75701, TX, USA; 3 Physics Department, Queensland University of Technology, Brisbane, Australia

**Keywords:** Quality assurance, Radiation field, Light field, Linear accelerator, Electronic portal images device, Computed radiography

## Abstract

**Background:**

The radiation field on most megavoltage radiation therapy units are shown by a light field projected through the collimator by a light source mounted inside the collimator. The light field is traditionally used for patient alignment. Hence it is imperative that the light field is congruent with the radiation field.

**Method:**

A simple quality assurance tool has been designed for rapid and simple test of the light field and radiation field using electronic portal images device (EPID) or computed radiography (CR). We tested this QA tool using Varian PortalVision and Elekta iViewGT EPID systems and Kodak CR system.

**Results:**

Both the single and double exposure techniques were evaluated, with double exposure technique providing a better visualization of the light-radiation field markers. The light and radiation congruency could be detected within 1 mm. This will satisfy the American Association of Physicists in Medicine task group report number 142 recommendation of 2 mm tolerance.

**Conclusion:**

The QA tool can be used with either an EPID or CR to provide a simple and rapid method to verify light and radiation field congruence.

## Background

The radiation field on most megavoltage radiation therapy units are indicated by a light field projected through the collimator by a light source mounted inside the collimator [1]. Depending on the manufacturer, this light source may be positioned at the location of the x-rays target by a rotating carousel or a sliding drawer assembly, or it may be positioned to one side of the collimator axis of rotation with the light reflected from a mirror. Traditional radiation therapy clinical practice involves aligning the treatment unit light field with the skin marks on the patient as the final confirmation that the patient is correctly positioned with respect to the radiation beam. It is therefore necessary that the light field agrees (is congruent) with the radiation field. No wonder that most recommendations for quality assurance of megavoltage radiation therapy equipment require checks of the degree of congruence of the light and radiation fields. A case in point is the American Association of Physicist in Medicine (AAPM) task group report no 40 (TG 40)- [2] and most recently report no 142 (TG 142) [3] of the same organization.

Traditionally films were used to verify that the light and radiation fields agreed with each other and with the indicated jaw settings. The developed film was visually compared with a drawing (or impression) of the light field on the film. Unfortunately, this method did not only prove tedious as each film envelope had to be marked up separately to the extent of necessitating the development of test tools to aid the procedure [4-6], it is equally been shown to be not very reliable [7]. Also with the drive toward film-less and chartless radiation therapy environment, there is a need to develop ways to carry out this important QA check. To this effect, there have been proposed techniques to use computed radiography (CR) system [8,9], photodiodes [10], diodes arrays and electronic portal imaging devices (EPIDS). So far, several test tools and appropriate software have been developed to quantitatively study light and radiation field congruence using EPIDS [4-6,11]. The use of some of the approaches is dependent on the availability of custom designed software used in a stand-alone fashion [5] or incorporated into the commercial EPID system [6]. However, these approaches involve potential cost and also in some cases access to the EPID software.

In this paper we report on the design and performance of a simple QA test tool which may be used with either EPID or CR to visually verify linear accelerator light and radiation field congruence. Quantitative assessment is possible and it can be carried out either using available EPID software (VARIAN) or a third party software like RIT (Colorado Springs, CO).

## Methods

### QA test tool

**Figure 1:**
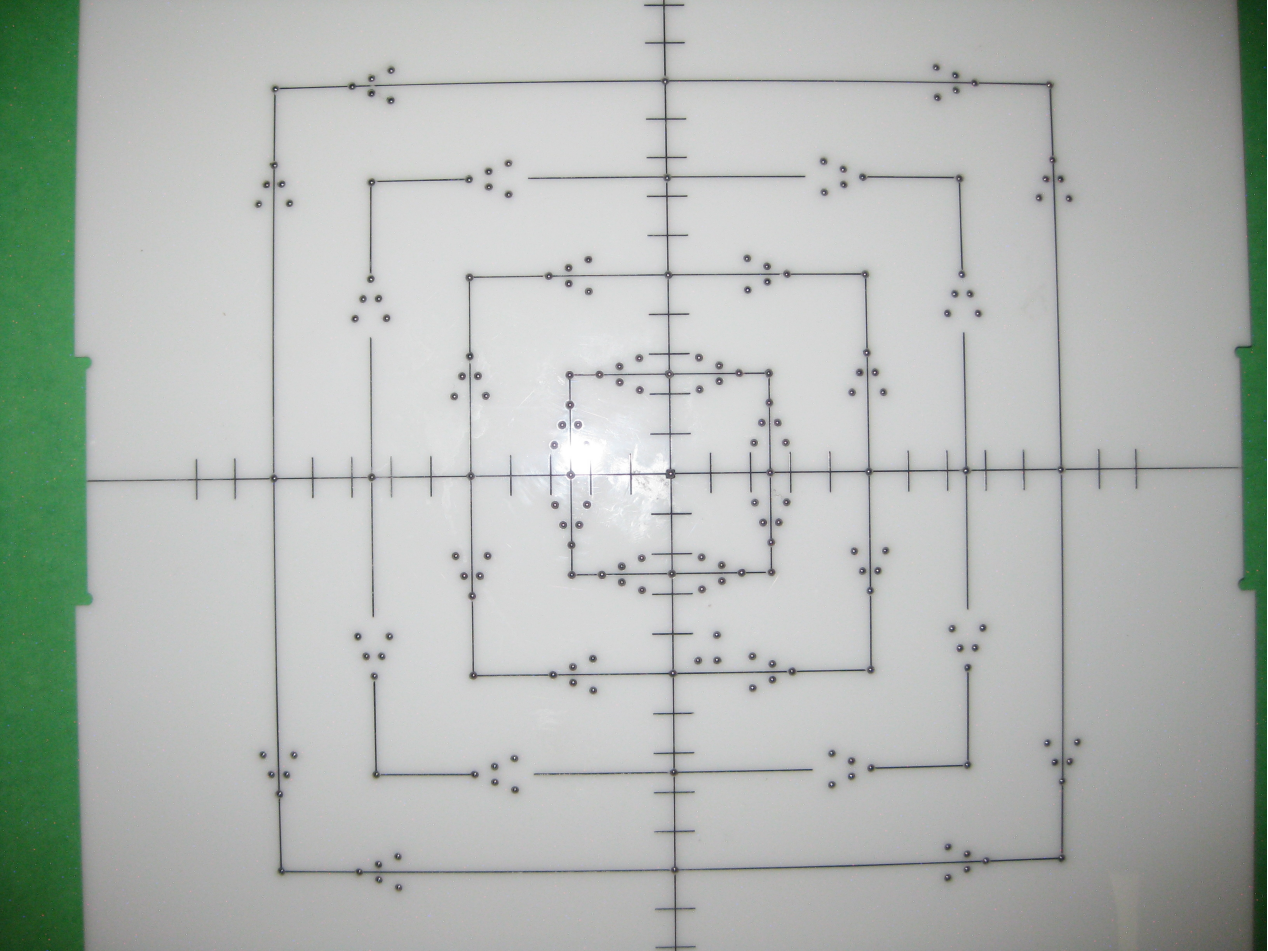
**A photograph of the light -radiation congruence QA test tool**.

The QA test tool is a modification of Isocentric Beam Checker II from Mick Radio-nuclear Instruments (Mount Vernon, NY). The QA tool consists of a large opaque acrylic screen backed by a secondary plate (Figure 1) (a secondary plate is not required if no film will be used). The screen is inscribed with lines precisely defining corners, edges and center of the screen's 2 mm × 2 mm, 5 cm × 5 cm, 10 cm × 10 cm, 15 cm × 15 cm and 20 cm × 20 cm fields. Intersecting center lines are inscribed with short lines spaced l cm apart. Tungsten markers of 2 mm diameter are embedded in the center and corners of the fields. These markers are also embedded on the sides of each field size, one at the exact field size, then 2 mm away and then 4 mm away, in the form of a V-shape (see Figure 1). When exposed, the tungsten markers project a sharp image on the EPID or film. Therefore, the necessity of pricking holes into the film is eliminated.

### Electronic portal imaging device (EPID)

In this study we used electronic portal imaging device (EPID) from Varian and Elekta.

### Varian aS1000

The Varian EPID (aS1000, Varian Medical Systems, Palo Alto, CA) consists of an electronic image acquisition system (IAS3) and image acquisition system software (PortalVision). The IAS3 contains an amorphous silicon (based on Gd_2_O_2_S:Tb detector) flat panel device mounted on a motorized robotic three axis exact arm and electronic for acquiring images. The robotic arm allows it to be positioned at source to EPID distance from 95 to 180 cm. It has an active imaging area of 40 cm × 30 cm (at focus to detector distance of 100). The image matrix is created from an array of 1024 × 768 photodiodes, giving an effective pixel size of 390 μm at FDD of 150 cm [12]. The maximum frame acquisition rate is 9.574 frames/second, the permitted dose range is 4 - 25 MV and the permitted dose rates are 50-600 mu/min [13].

The aS1000 EPID can be operated in various image acquisition modes that are comprised of a combination of beam energy, repetition rate, and scanning mode. These acquisition modes include low quality mode and high quality mode. The image is an average signal of a definite number of frames, with the low quality mode averaging 2 frames and high quality mode 4 frames. Medical linear accelerators have an integral dose rate servo (DRS) unit whose function is to maintain a steady dose rate. For Varian dual-energy LINACs, the servo accomplishes this by adjusting the length of the beam pulse. To avoid variations in portal image intensity arising from intentional pulse-to-pulse adjustments of beam fluence, the DRS is switched off during image acquisition.

### Elekta iViewGT

The iViewGT from Elekta consists of a Perkin Elmer Amorphous Silicon detector mounted in a retractable arm assembly. The panel radiation sensitive layer has a size of 41 cm × 41 cm and consists of 1024 × 1024 detector elements with a pitch of 0.4 mm and 16-bit pixels. The mechanical arm holding the detector consists of two stages that locate the detector in the position to acquire images. The detector can be moved in the longitudinal and lateral directions. It is made of the following layers: Aluminum top cover, air gap, 1 mm thick copper plate, graphite layer, LANEX Fast terbium-doped gadolinium oxysulfide phosphor screen (Gd_2_O_2_S:Tb), attenuating film and a-Si:H photodiode array [14,15]. Image data is read between the radiation pulses, which is synchronized by the PPG PCB and controlled by a Detector Control Board (DCB). A combination of software and hardware triggering is used to capture the image. Image data is read from the panel through a data link into a frame grabber in the iViewGT computer.

iViewGT can create a high quality image with as little as 1 MU and is available on screen within a fraction of a second. Images can be displayed in a variety of ways for optimum review. They can be further enlarged, scaled, measured, flipped and rotated, as well as enhanced using the CLAHE* (Contrast limited adaptive histogram equalization) feature.

### Linear accelerator (LINAC)

We tested this tool on two Varian machines (Varian Medical Systems, Palo Alto, CA): 21IX and Novalis TX and two Elekta Precise machines (Elekta Inc, Norcross, Ga). The Varian machines are dual-energy machines delivering 6 - and 18 -MV photon beams. The Elekta Machines are triple-energy machines delivering 6 MV, 10 MV and 18 MV photon beams.

### Kodak computed radiography

Computed Radiography (CR) is a digital imaging system that has been introduced into radiotherapy for megavoltage portal imaging [16]. CR is based on the principle of Photostimulable Luminescence (PSL). The active layer of a CR plate is a coating of BaSrFBr doped with Eu in a +2 ionic state. When exposed to ionizing radiation, the energy absorbed by the plate is stored in a semi-stable state [17] consisting of trapped electron hole pairs in the BaSrFBr:Eu2+ phosphor grains. When the plate is scanned within the CR digitizer by a focused He-Ne laser beam, the trapped electrons are locally excited and recombine with the trapped holes to emit light. The quantity of light is proportional to the density of trapped electron/hole pairs and thereby to the locally absorbed x-ray dose. The emitted photons are collected by a photomultiplier tube (PMT) that converts the luminescence into an electrical signal [18]. This signal, logarithmically amplified and correlated with the scanning laser spot position, is digitized by means of an analog-digital (A/D) converter and sent to a computer for the reconstruction of the 2-dimensional image. A strong fluorescent lamp erases any remaining information in the image plate so that it can be reused. When performing MV dose related procedures, the scanner is operated in port film mode (photomultiplier tube gain reduced by a factor of 100 when compared to diagnostic mode) using a resolution of 1024 or 2048 (user selectable) pixels distributed across the width of the CR plate.

### Measurement: varian technique

The detector (EPID) was brought to 150 cm (source to detector distance (location p2)). The QA tool was placed at isocenter. The placement of the QA tool would impact the acquired image. For example if placed on the tennis racket portion then that racket will be visible on the acquired image. The Novalis TX has an Exac Trac imaging couch making it possible to place it directly on the couch since this couch is designed to facilitate on board imaging. On the IX, the QA tool was placed on the rails so that the tennis racket was not visible in the image. We acquired the images using two techniques - clinical mode and service mode. In service mode, we used the AM (acquisition maintenance) application, but in clinical mode, the double exposure technique was employed.

### Image acquisition protocol: clinical mode

A phantom is set up as a patient and is called up in the record and verify software (in one of our centers we use Mosaiq (Elekta). Double exposure portal image is added to the images.

1. The gantry, collimator and couch were set to 0 degree (IEC standard)

2. The QA tool was set at isocenter in the treatment room with the light field set to match the field edged on the QA tool.

3. The EPID was brought out so that focus to detector distance was 150 cm

4. An image was then acquired for the appropriate field size (the physical or console set field size was verified against the dosimetric field size using a graph paper)

5. The second exposure was then acquired with the field size increased. When using a double exposure sequence template the 4DITC automatically sequences to the open after the first image has been acquired

6. Lastly, edge detection algorithm was used to detect the field edges

True congruence is achieved if the detected field size matches the first marker on the V-shaped tungsten inserts.

### Image acquisition protocol: service mode

1. The gantry, collimator and couch were set to 0 degree (IEC convention)

2. The QA tool was set at isocenter in the treatment room with the light field set to match the field edged on the QA tool.

3. The EPID was brought out so that focus to detector distance was150 cm

4. The linac was put into service mode and the energy, and the dose rate were selected

5. The AM software was opened

6. The EPID could be calibrated if required by acquiring a dark image and a flood image

7. The image acquisition mode was chosen

8. The image was acquired

9. The edge detection feature from the image analysis menu was used to find the edges

Congruence is achieved if the detected field size matches the first marker on the V-shaped tungsten inserts

### Measurement: elekta image acquisition protocol

The phantom was placed at isocenter (SSD = 100 cm) directly on the carbon fiber couch. 2 MU each were delivered, for different field sizes, and the images directly acquired on the EPID using the iViewGT software version 3.2. Image acquisition can be done in three different modes; single-exposure mode, double-exposure mode and movie-exposure mode. Also deliberate errors in field sizes were introduced to simulate a realistic condition when the field sizes defined by the MLC and jaws actually have a small discrepancy. The images of the test tool, directly showed the magnitudes and directions of the arbitrary errors introduced. For better visualization in double exposure mode, it is preferable for the open field exposure to be carried out before the treatment field.

### Film measurement

A therapy verification film (Kodak, X-Vmat, Eastman Kodak Company, Rochester, NY) was used to validate the EPID measurements. The film was placed on a solid water and 2 cm buildup. The film was developed by means of an automatic procedure. No specific calibration was made; however, the film was exposed to a dose value to guarantee to be in the dose-density linear region of the HD curve.

### Interpretation of the images

PortalVision and iViewGT have various tools to manipulate the image. However, the most useful tool is the manual Window/Level, where the contrast and brightness can be manipulated for better visualization of the markers. For PortalVision, in both clinical and service mode, there is also an option to use edge detection algorithm to automatically determine the edge of the radiation field.

### Visual validation

**Figure 2:**
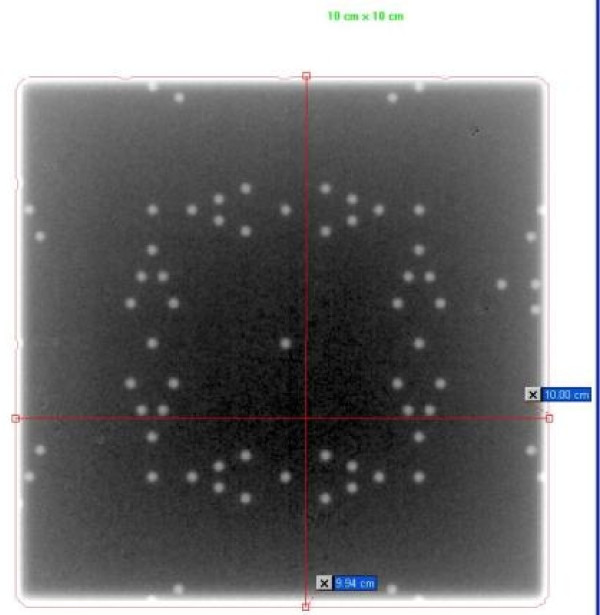
**Light and radiation field congruence for a 10 cm × 10 cm field size of Varian TX linac using a single exposure technique in service mode with Varian Portal Vision**.

The tool is setup at 100 cm SSD on the table and aligned with the light field set according to machine parameters. The size of the light field can be verified using other tools (rulers or graph paper). A single exposure of a 10 × 10 field at Isocenter is produced (Figure 2). Then, one looks at the V-shaped markers at the edge of the field. It is worth noting that the total possible number of markers that can be visible on the EPID is 5. The marker radius is 1 mm, hence the accuracy of the visual reading is limited to 1 mm. The field size can be read with a range of ± 5 mm of the set field size. The following conclusions are possible:

1. If two full markers are visible inside of the radiation field edge then the field is congruent within 2 mm on that side with the radiation field smaller than the light field.

2. If the third marker (corresponding to the corner of the V) is visible partially or fully the conclusion is that the field congruency is within 1 mm on that side.

3. If part of the second circle on the outside of the field edge is visible then the field congruency is still within 2 mm on that side but the radiation field is larger than the light field.

4. If the second marker on the outside is fully visible then the congruency could be 3 mm. on that side.

Cases 1, 2, and 3 give passing results according to TG-142 specifications of 2 mm or 1% on a side.

It is possible to produce the images using a double exposure technique. We will talk about this approach in the discussion section.

Therefore even in the absence of edge detection software, this QA tool is still useful for visual validation of light field. One should have predetermined a good combination of window and level setting corresponding to the edge being equivalent to the 50% of the central axis dose. This is done in the following manner: The tool is set at Isocenter with film under it and on top of "plastic water" for backscatter. The film is irradiated at the same time as the EPID. The film is processed and the window and level necessary to replicate the film on the EPID is discovered and then used for subsequent checks.

### Quantitative validation

The tool is set up as before and the image is sent to RIT. The software for light field versus radiation field analysis can be run using the settings for IsoALign. The software provides quantitative results of the congruency between radiation field and light field. We ran tests using the RIT software that helped us prove to ourselves that the visual interpretation is correct. Additionally we had an engineer from the Elekta company use the tool and EPID to adjust the congruency of light and radiation fields. Then we took films the old fashioned way to verify the accuracy of our tool for this endeavor. The results were within 1 mm on each side for the 10 × 10 cm field size.

## Results

**Figure 3:**
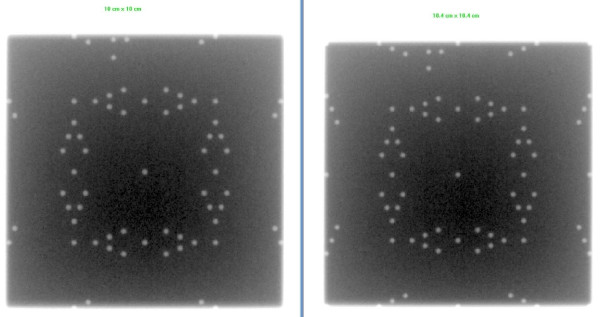
**Varian Portal Vision is used to acquire a single exposure in service mode for different field sizes 10 cm × 10 cm field size compared to a 10.4 cm × 10.4 cm**.

**Figure 4:**
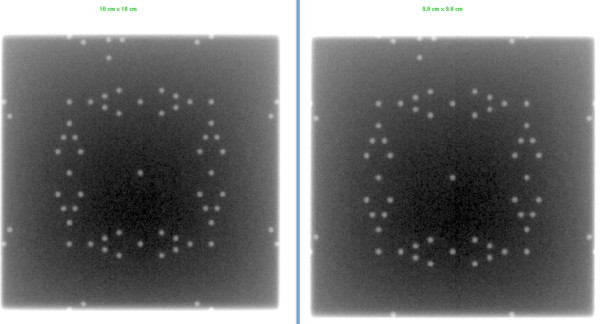
**Varian Portal Vision is used to acquire a single exposure in service mode for different field sizes 10 cm × 10 cm field size compared to a 9.8 cm × 9.8 cm**.

**Figure 5:**
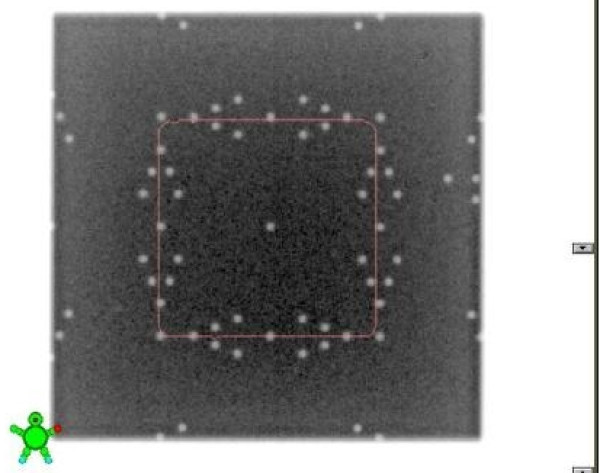
**Light and radiation field congruence for a 10 cm × 10 cm field size of Varian TX linac using a double exposure technique in service mode with Varian Portal Vision**.

**Figure 6:**
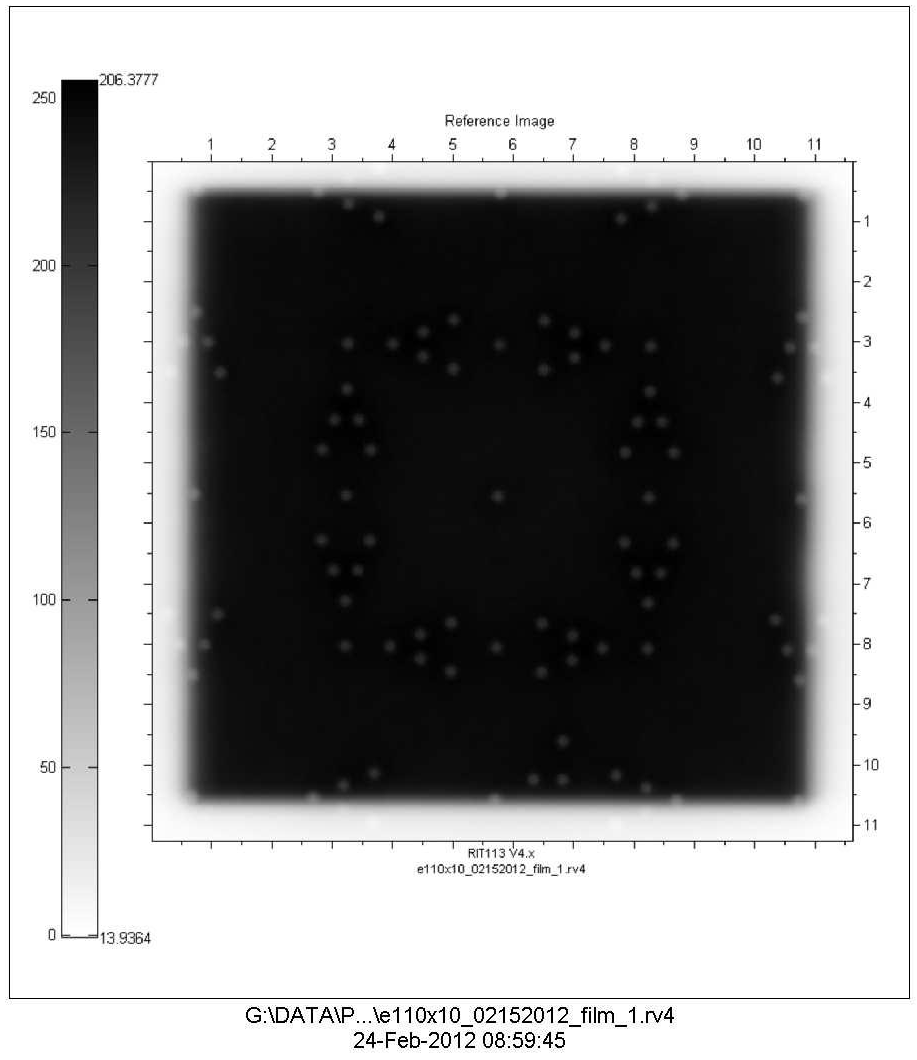
**Light and radiation field congruence for a 10 cm × 10 cm field size of Elekta Precise linac using a single exposure technique in service mode with iView GT**.

**Figure 7:**
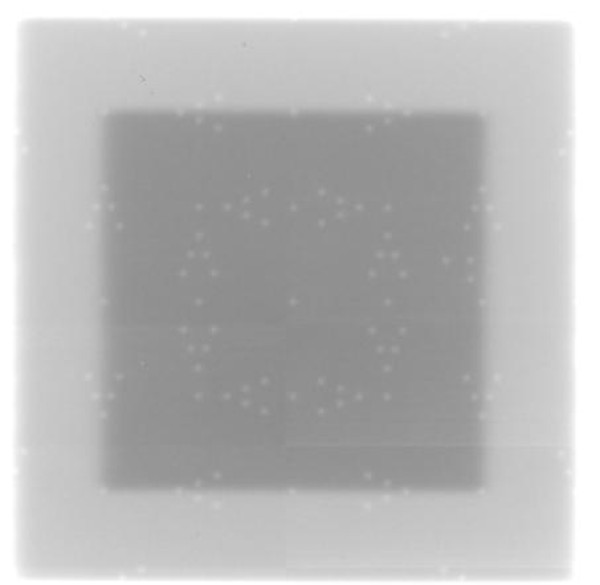
**Light and radiation field congruence for a 10 cm × 10 cm field size of Elekta Precise linac using a double exposure technique in clinical mode with iView GT**.

**Figure 8:**
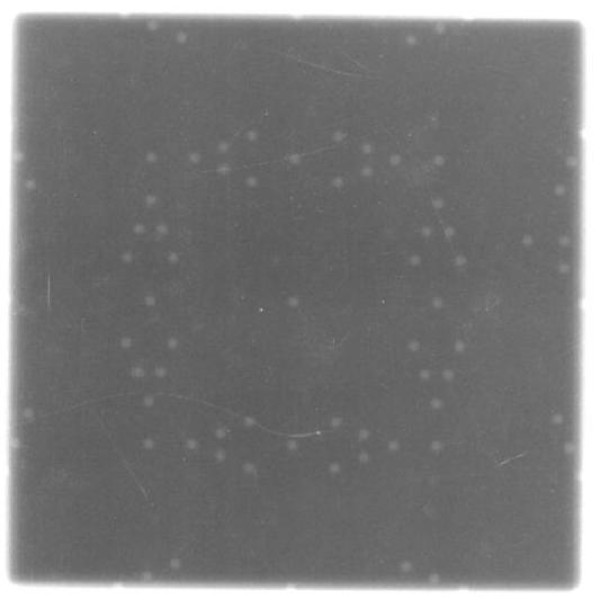
**Light and radiation field congruence for a 10 cm × 10 cm field size of Varian TX linac using a single exposure technique with Kodak CR**.

**Figure 9:**
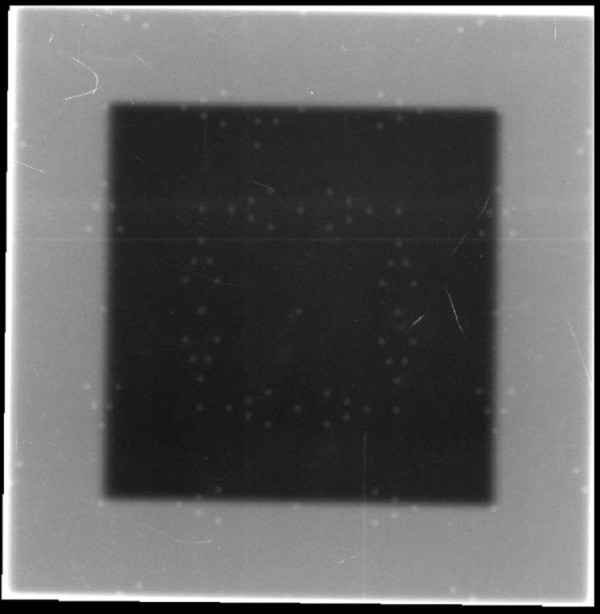
**Light and radiation field congruence for a 10 cm × 10 cm field size of Varian TX linac using a double exposure technique with Kodak CR**.

This simple QA tool is very light, portable and can be used with any EPID device and CR system. It was designed such that each light field could be aligned with the scale on top of the phantom. Images acquired on Varian EPID using the single and double exposure technique are presented in Figures 2, 3, 4, 5. Images acquired on Elekta iViewGT using the single and double exposure technique are presented in Figures 6 and 7. Images acquired with variable field sizes, to determine to minimum detectable field changes are presented in Figures 3 and 4. Images acquired on Kodak CR using the single and double exposure technique are presented in Figures 8 and 9 As can be seen in Figure 5, the PortalVision image detection algorithm successfully detected the edges of the field which also coincided with the first marker of the V-shaped markers designed. The accuracy of the edge detection was verified to be within 0.01 mm (comparing the dosimetric field size with the edge detection determined field size)

**Figure 10:**
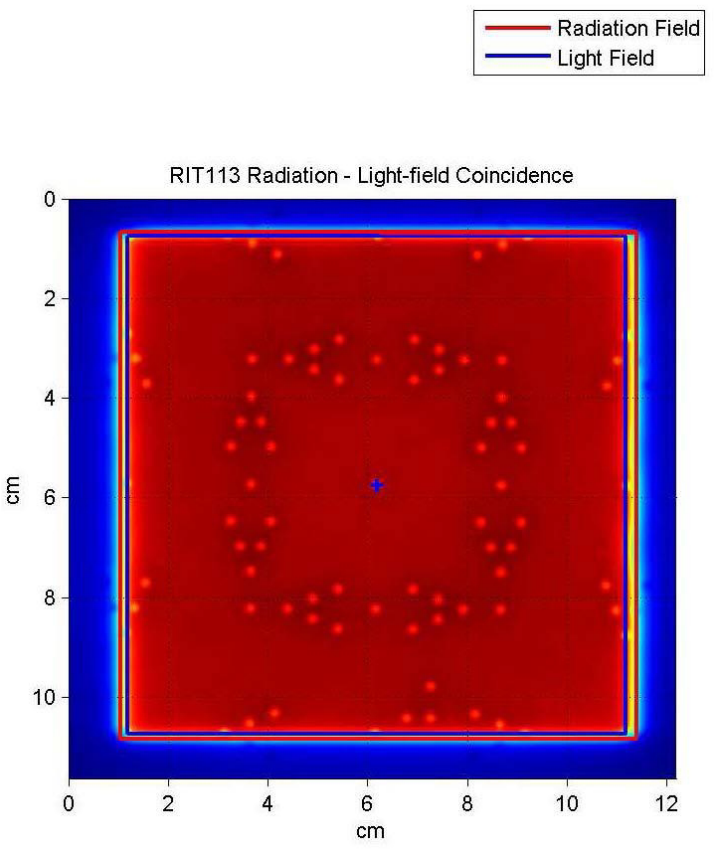
**Light radiation field congruence of an iViewGT image analyzed using RIT software**.

**Figure 11:**
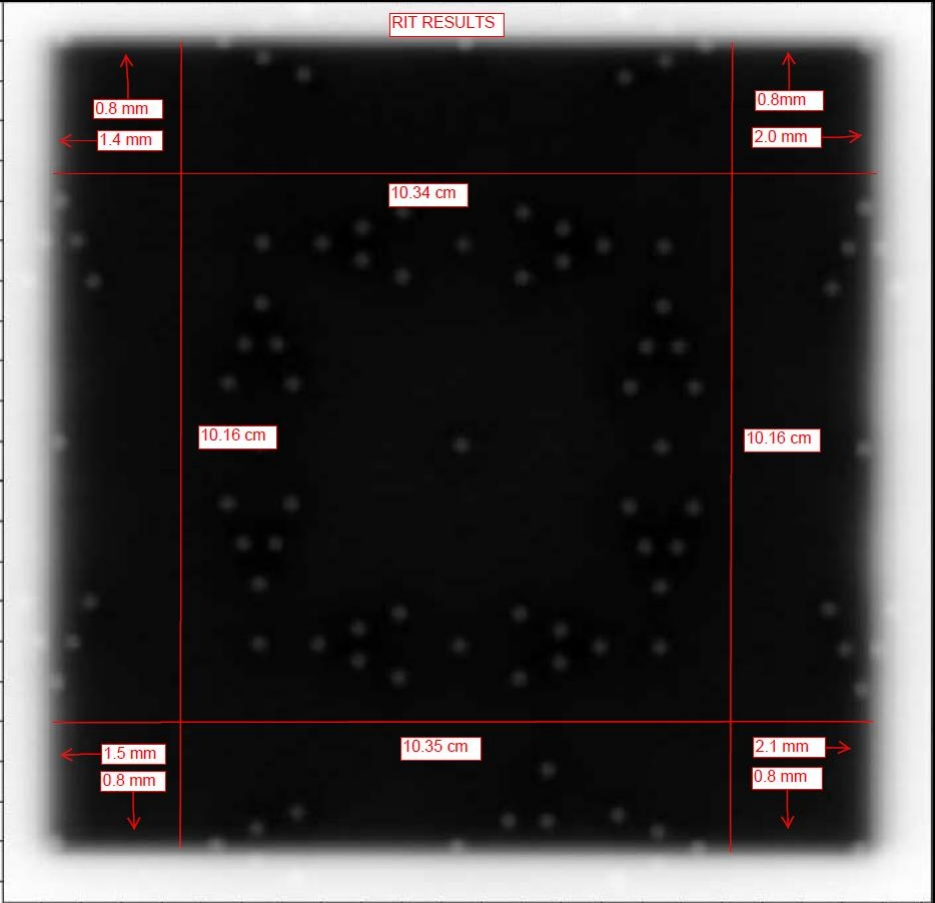
**Quantitative results of the light radiation field congruence of iViewGT image analyzed using RIT software**.

We carried out tests using the RIT software that validated that our visual interpretation was correct. The results of this quantitative analysis are presented in Figures 10, and 11. Figure 10 is a copy of the image with the results incorporated and Figure 11 shows the light field and radiation fields as interpreted by the RIT software. In Figure 11 for example, at the upper left corner of the image the box with the arrow pointing up indicates the result for the top-left corner being 0.8 mm, the report indicates 0.78918 mm. In a visual inspection of this image one would have used the process indicated in H1 above: since one can see two of the markers and part of the third marker the field edge is within 1 mm of the light field (H1.2) A quick look at all the results indicated in figure (11) is enough to convince ourselves that through visual inspection one can discern how good or bad the light versus radiation congruency is for the field in question. As a further test we engaged our service engineer from Elekta to use the tool and EPID to adjust the congruency of light and radiation fields. Then we took films the old fashioned way to verify the accuracy of our tool for this endeavor. The results were within 1 mm on all sides for the 10 × 10 field size.

Varian PortalVision has different image acquisition modes. We tested the low quality and high quality modes and found that there were no significant differences between them when the tool was used directly on the couch. Since no significant attenuation is encountered no significant difference is observed between the low image and high quality images. However, when a build up is placed on the QA tool, then a visible qualitative improvement image can be observed between the high quality and low quality image acquisition mode.

The EPID is required to be calibrated regularly and the calibration is designed to remove background noise and provide a spatially uniform response for clinical imaging. The response of the EPID is dependent on the photon energy and the repetition rate of the radiation beam. Hence, calibration should be performed separately for each acquisition mode. The Varian EPID calibration involves acquiring a dark-field image and a flood-field image. A dark-field image provides information about background noise and is obtained by taking a base reading for each pixel in the absence of radiation. The test of light field and radiation field alignment was qualitative in nature. Hence there is no need for EPID calibration prior to measurement. We took images before and after calibration and no qualitative effect was observed.

## Discussions

The results from performing this study with both sets of machines at our two centers are very promising. As part of our monthly QA program to ensure the alignment of the light field with the radiation field per TG 40/142, physicists at our respective centers spend a considerable amount of time, setting XV films on the couch, developing the films, and then analyzing the results using RIT or other analysis software. Indeed this has been a cumbersome process; involving multiple films if adjustments to the field sizes, or the light and radiation field are needed. This test tool obviates the necessity to shoot multiple films, as the results are almost instantly visible, thereby enabling a quicker quality control process. The introduction of deliberate errors of various magnitudes and directions as described above is easily visualized on the EPID images, thereby enabling the physicist to make the necessary corrective changes in real time.

The double exposure technique provided a better approach for visualization of the congruence. It is easy to count the visible markers in the image. However, for PortalVision (PV) the contrast between the two images was not significant, partly because only 1 MU was used for each exposure. Nonetheless, using the intensity profile the edge detection algorithm was able to detect the radiation field and it coincided with the light field as shown by the tungsten markers. Contrary to the PV, the double exposure technique with the iViewGT showed a significant contrast between the two images and hence the agreement could be evaluated without the need for edge detection (Figure 7).

With the wide availability of EPIDs and also with most departments going filmless, this QA tool provides a very convenient way to check radiation and light field congruence. It is inexpensive easy to use, quick to setup and the results are instantaneous. Most of the available tools have only one field size. Our QA tool has 4 different field sizes making it possible to test a variety of field sizes.

Previous studies have already demonstrated the feasibility of EPID for QA [19,20] and especially light field and radiation congruence [4-6,11]. Dunscombe et al. [4] and Luchka et al. [5] have previously explored light and radiation congruence with EPID. However, they used video based system that has poorer image resolution compared to the amorphous silicon detector reported herein. Imaging systems based on amorphous silicon produce images with a higher detective quantum efficiency (DQE) than fluoroscopic imaging devices because much more optical photons are detected [21].

Also, other test tools have been developed and tested by different researchers [4-6,11]. However, these test tools have different limitations and are not widely available. We have evaluated a test tool here that is commercially available. Also we have not used any in-house software for analysis making it possible for any center with an EPID to purchase the tool and be able to apply the technique.

The CR plate provides another approach to verify the light/radiation congruence for centers that do not have EPID. CR has a few advantages over traditional films. For example, the possibility of digital post processing of CR overcomes poor tissue contrast which was the major limitation of conventional radiographic portal film [17]. Other advantages of CR include: It is economical because of low running costs compared to film, a low initial investment compared to the EPID and possibility of using one CR system for several therapy units. Secondly the similarity of photostimulable phosphor plate to film gives it an edge over the EPID which is also limited by its radiosensitive electronics [8]. Finally in this digital age, another advantage of using CR or EPID for light radiation field congruence is the ability to store images online and have permanent record.

Other researchers such as Peace et al. [8] and Soh et al. [9] have demonstrated the successful use of CR for LINAC QA. Peace et al. [8] designed a test tool for light field radiation congruence by embedding a 1 mm diameter lead wires in a Perspex. They used a single technique method and found that the lead wires enclosing the field in the cross-plane could not be distinguished very clearly from the unexposed part of the image. They then suggested the contrast of the lead wire in the image could be improved by performing a double exposure. We used a double exposure technique and found a good contrast with the tungsten markers. To check the coincidence between light field and radiation field, Soh et al. [9] placed four coins at each of the four edges of a square light field size (15 cm × 15 cm). They found the CR could be used even though their approach was very simplistic.

One of the main limitations that have been put forward when using visual assessments is that the process is extremely subjective. Also, that the results could depend on the viewing conditions, monitor performance and observer experience [4]. With this simple QA tool, setting a baseline, contrast, the process can be easily reproduced and the subjectivity is eliminated and also the tungsten markers have a large contrast compared to the acrylic and thus would be very visible under different conditions.

Our study has its limitations. For example, we did not use the tool to test the radiation/light match over a period of time. However, there is no reason to believe that slight changes in the performance of the EPID over a period of time should affect the results. It is expected that with proper calibration of the EPID, the QA tool could be used at any time. Also the QA tool is not designed to fit on the LINAC head and hence to test in different gantry angles can be problematic. But then since it is only a plate and the EPID rotate with the gantry, the QA tool can easily be adjusted to the appropriate gantry angle.

## Conclusions

For the safety of patient treated with radiation, it is necessary to ascertain that the light field corresponds to the radiation field. It is evident from the preceding paragraphs that the quality assurance (QA) tool evaluated in this paper can be used for monthly QA, to verify the light and radiation field congruence using either EPID or CR. One can visually detect changes in radiation and light field congruence of less than 2 mm which will meets AAPM TG 142 tolerance. Either single exposure or double exposure technique can be used with acceptable results. This has been validated using both PortalVision from Varian, IViewGT from Elekta and CR from Kodak.

## Competing interests

The authors declare that they have no competing interests.

## Authors' contributions

CFN designed the QA tool, the experiment, collection of the data, drafted the manuscript. BC and PD participated in the design of the experiment, collection of data and drafting of the manuscript. All authors approved the final version of the manuscript.

## Authors' information

Dr. Njeh obtained his Ph.D. degree in Medical Physics from Sheffield Hallam University, U.K. after graduation; he worked at Addenbrooke's Hospital in Cambridge, U.K. and Queen Elizabeth's Hospital in Birmingham, U.K. He then went to the US as a Visiting Postdoctoral Fellow at the University of California, San Francisco, CA where he was subsequently appointed an Assistant Professor of Radiology. He later completed a Medical Physics Residency at Johns Hopkins University, Baltimore, MD and. Dr. Njeh is certified in Therapeutic Radiologic Physics by the American Board of Radiology (ABR). Dr Njeh is a member of the American Association of Physicist in Medicine (AAPM) and the American Society of Radiation Oncology (ASTRO). He is member of the minority recruitment at AAPM and education community at ASTRO. Dr. Njeh is a manuscript reviewer for a number of international journals including: Medical dosimetry, Physics in medicine and biology and Osteoporosis international. Dr Njeh is the author of over 60 peer reviewed articles, more than 10 book chapters and 2 books. His research interests include image guided radiation therapy, linac QA and accelerated partial breast irradiation.

Dr. Caroprese obtained a Ph.D. in theoretical physics at UCLA, after a few years became interested in Medical Physics and entered the field trough an internship at Columbia Regional Hospital and the University of Missouri at Columbia. He later served as advisor for a Master Level Thesis at the University of Missouri, Columbia besides other teaching duties. Dr. Caroprese is certified by the American Board of radiology (ABR) and is a member of AAPM and ASTRO. Dr. Caroprese has spent his entire career as a Medical Physicist working in clinical environments. Dr. Caroprese has been dedicated to Quality Assurance, Commissioning, Clinic design and implementation of new techniques besides the day to day clinical aspects of the job (IMRT, SRS, SBRT, HDR, LDR)
